# Ample glycosylation in membrane and cell envelope proteins may explain the phenotypic diversity and virulence in the *Mycobacterium tuberculosis* complex

**DOI:** 10.1038/s41598-019-39654-9

**Published:** 2019-02-27

**Authors:** Alemayehu Godana Birhanu, Solomon Abebe Yimer, Shewit Kalayou, Tahira Riaz, Ephrem Debebe Zegeye, Carol Holm-Hansen, Gunnstein Norheim, Abraham Aseffa, Markos Abebe, Tone Tønjum

**Affiliations:** 1 Department of Microbiology, University of Oslo, PO Box 4950, Nydalen, NO-0424 Oslo Norway; 2 Addis Ababa University, Institute of Biotechnology, PO Box 1176, Addis Ababa, Ethiopia; 3 International Center of Insect Physiology and Ecology (ICIPE), P.O. Box 30772-00100, Nairobi, Kenya; 4 Centre for Applied Biotechnology, Uni Research Environment, Bergen, Norway; 5 Coalition for Epidemic Preparedness Innovations (CEPI), P.O. BOX 123, Torshov, 0412 Oslo Norway; 6 Infection Control and Environmental Health, Norwegian Institute of Public Health, PO Box 4404, Nydalen, NO-0403 Oslo Norway; 7 Armauer Hansen Research Institute, Jimma Road, PO Box 1005, Addis Ababa, Ethiopia

## Abstract

Multiple regulatory mechanisms including post-translational modifications (PTMs) confer complexity to the simpler genomes and proteomes of *Mycobacterium tuberculosis* (Mtb). PTMs such as glycosylation play a significant role in Mtb adaptive processes. The glycoproteomic patterns of clinical isolates of the *Mycobacterium tuberculosis* complex (MTBC) representing the lineages 3, 4, 5 and 7 were characterized by mass spectrometry. A total of 2944 glycosylation events were discovered in 1325 proteins. This data set represents the highest number of glycosylated proteins identified in Mtb to date. O-glycosylation constituted 83% of the events identified, while 17% of the sites were N-glycosylated. This is the first report on N-linked protein glycosylation in Mtb and in Gram-positive bacteria. Collectively, the bulk of Mtb glycoproteins are involved in cell envelope biosynthesis, fatty acid and lipid metabolism, two-component systems, and pathogen-host interaction that are either surface exposed or located in the cell wall. Quantitative glycoproteomic analysis revealed that 101 sites on 67 proteins involved in Mtb fitness and survival were differentially glycosylated between the four lineages, among which 64% were cell envelope and membrane proteins. The differential glycosylation pattern may contribute to phenotypic variabilities across Mtb lineages. The study identified several clinically important membrane-associated glycolipoproteins that are relevant for diagnostics as well as for drug and vaccine discovery.

## Introduction

Tuberculosis (TB) is a major threat to public health, causing more than three deaths per minute globally. The causative agent is *Mycobacterium tuberculosis* (Mtb), and the TB crisis is exacerbated by the emergence of multi-drug-resistant (MDR) and extensively drug-resistant (XDR) Mtb strains. This situation highlights the urgent need for a comprehensive understanding of virulence and pathogenicity determinants of the *M*. *tuberculosis* complex (MTBC) to pave the way for the development of alternative TB control and prevention. Post-translational modifications (PTMs) including protein glycosylation are crucial in this regard. The unique Mtb glycoconjugates in the cell envelope are the predominant basis for host–pathogen interactions, antigenicity, and virulence determination and constitute one of the major components causing antimicrobial resistance (AMR) in Mtb^1–6^. Glycosylation in Mtb has mainly been detected in surface-exposed proteins and in some other membrane proteins^7^. The unique structure, antigenicity and essentiality of Mtb cell envelope glycoconjugates for mycobacterial growth provide opportunities for the development of novel drugs, vaccines, diagnostics and biomarkers^1^.

Mtb glycoproteins play a critical role in a number of biological activities including cell adhesion and invasion, protein stability, localization, and maintenance of protein conformation^8–12^. Other functions influenced by glycosylation are cellular signaling, AMR, immunomodulation, intracellular bacterial survival, biofilm formation, protein complex formation, antigenicity, pathogenicity and virulence^8–12^. Recently, it has been shown that protein glycosylation was associated with low cell envelope permeability and AMR in the multi-resistant *Mycobacterium abscessus*^13^. Furthermore, glycosylation protects proteolytically-sensitive cleavage sites, thereby maintaining the membrane-association of the protein by its lipid anchor, and may also be linked to protein export^10,14^.

The Mtb cell envelope is composed of an inner plasma membrane, a cell wall core with an outer mycomembrane, and an outermost layer, known as the capsule composed of polysaccharides, lipids and proteins^1^. The cell wall core is composed of peptidoglycan (PG) covalently linked via phosphoryl-N-acetylglucosaminosylrhamnosyl to arabinogalactan (AG), which in turn is esterified to α-alkyl, β-hydroxy long-chain mycolic acids, forming the mycolyl arabinogalactan-peptidoglycan (mAGP) complex^1,15^. This complex is essential for bacterial viability and is the basis of susceptibility and resistance to many anti-TB drugs including ethambutol (EMB) and ethionamide (ETH)^12,16^. Mannose-capped lipoarabinomannan (LAM), one of the key Mtb virulence factors, is a surface-exposed lipoglycan anchored to the inner and outer membranes via a mannosyl phosphate inositol^17^. The soluble components of the cell envelope include free lipids, proteins, LAM, and phosphatidylinositol mannosides (PIMs), which are signaling effector molecules in bacterial pathogenesis and disease processes^15^. The nature and amounts of the mycomembrane and capsular material vary among Mtb isolates and is likely to impact significantly on the pathogen phenotype and outcome of the pathogen-host interaction^9,18^.

PG is a polymer of alternating N-acylated muramic acid (MurNac) and N-acetylglucosamine (GlcNac) residues linked in a β (1 → 4) configuration with cross-linked peptides of varying composition attached to the muramyl moieties^19,20^. PG glycosyltransferases use the lipid-linked donor precursor for the synthesis of oligo-β-(1 → 4)-[GlcNAc-β-(1 → 4)-MurNAc(peptide)] glycan strands^21^. In contrast to most other bacteria, muramic acid moieties are N-glycolylated (oxidized) in mycobacteria^15^. In Mtb, MurNGly, MurNAc and Mur residues are present in the precursor pool and in the PG^20^.

The membrane-bound glycosyltransferases or oligosaccharyltransferases (OSTs) catalyze the transfer of the monosaccharide moiety of an activated nucleotide-sugar substrate from lipid carriers to acceptor substrates, such as monosaccharides, oligosaccharides, proteins, lipids, small organic molecules, and DNA, and hence produce a wide variety of biomolecules^8,22^. Glycosidases are enzymes involved in both the degradation of glycans and the removal of monosaccharides to form intermediates that are acted upon by glycosyltransferases for the biosynthesis of glycans^22^.

*Campylobacter jejuni* and *Neisseria meningitidis* have well-characterized bacterial N-linked and O-linked glycosylation systems, respectively^23^. In N-linked protein glycosylation, an oligosaccharide is transferred by N-OST from a lipid donor to asparagines (N) located within the well-recognized consensus sequence D/E-Y-N-X-S/T and N-X-S/T (Y≠P, X≠P) of proteins^24,25^. Bacterial O-OSTs are responsible for the reversible attachment of glycans to hydroxyl groups of serine (S), threonine (T) and tyrosine (Y) residues, with no apparent sequence specificity^26,27^.

Glycoproteomics is likely to identify Mtb virulence factors because glycoproteins on the bacterial cell envelope are used by mycobacteria to enable their entry into the primary human host cell, the macrophage^28^. It has been proposed that Mtb interacts with mannose receptors (MRs) on host cells via mannosylated proteins to enter the macrophages^29^. Despite the vital importance of these proteins in Mtb pathogenesis, our current knowledge of Mtb glycoproteins is still limited, and only a few secreted and cell wall-associated glycoproteins have been described to date^8,28,30,31^. Previous studies have used laboratory strains as model systems to study glycosylation in Mtb. However, only a few sub-groups within the genetically conserved MTBC appear to cause extensive outbreaks with different clinical presentation and AMR^32–35^. In this study, we employed qualitative and quantitative mass spectrometry and bioinformatics to explore the glycoproteomic patterns of clinical isolates from four lineages of the MTBC, lineages 3, 4, 5 and 7, to investigate the role of protein glycosylation in Mtb adaptation, survival and AMR.

Our study reveals the presence of a number of glycoproteins that play roles in MTBC virulence and pathogenesis. These include proteins involved in pathogen-host interaction, transport and biosynthesis of MTBC cell envelope components, and drug efflux pumps, which are attractive pharmacological targets. Furthermore, we found quantitative differences in glycosylation patterns among the different lineages of MTBC that may potentially contribute to explaining their phenotypic characteristics.

## Results

### Abundance of both O- and N-glycosylation profile among members of the MTBC

**Figure 1:**
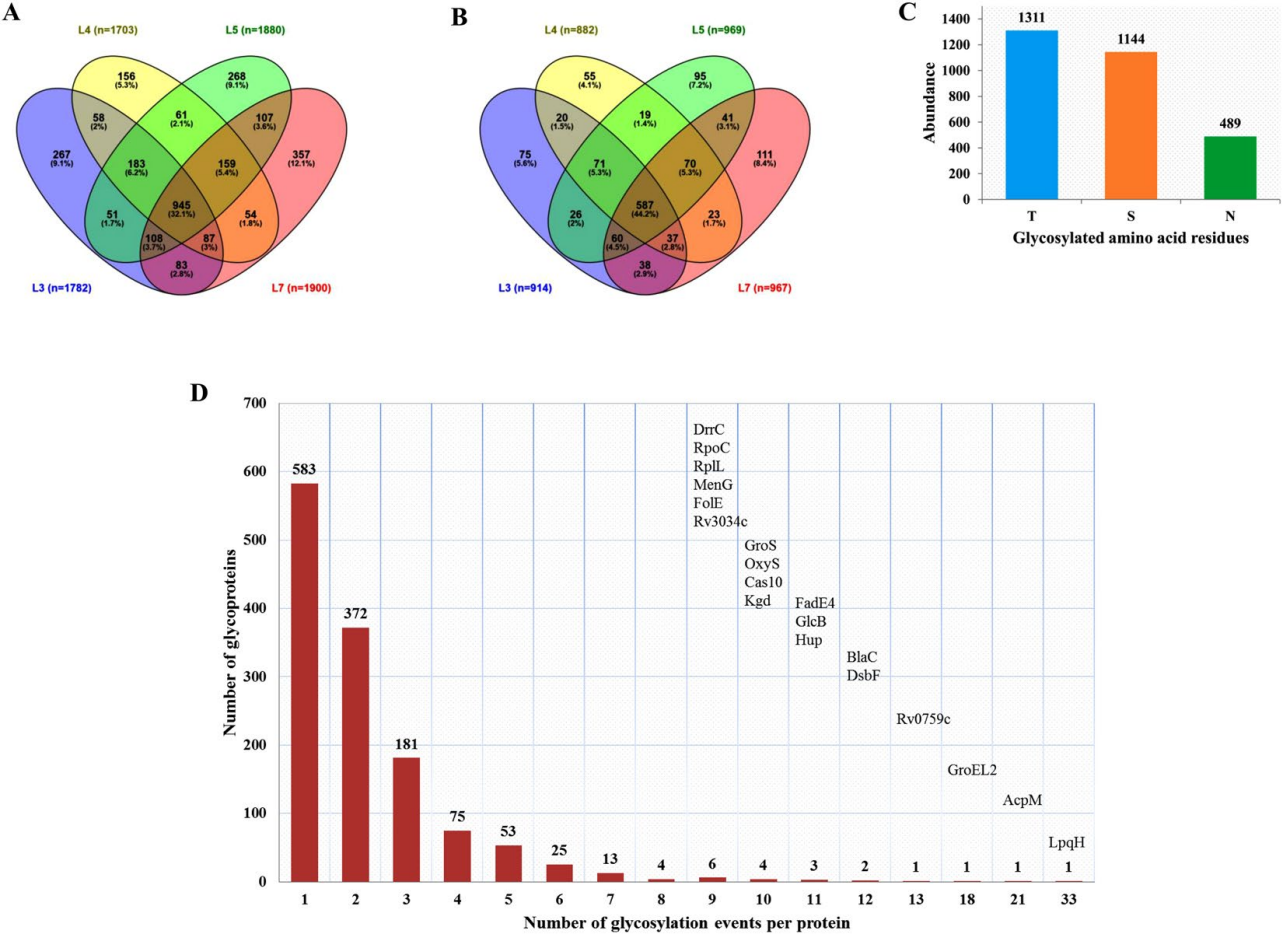
Abundance of glycosylation and glycoproteins in MTBC. Venn diagram showing the number of glycosylation events identified among the four lineages (N = 2944) (**A**), and the number of glycoproteins identified among the four lineages (N = 1325) (**B**), the number of N- and O-glycosylation events identified (N = 2944) (**C**), and number of glycosylation events identified per individual glycoprotein (**D**).

After filtering the data for potential contaminants and hits to the reverse database, the peptide intensities were log2-transformed. For protein identification, the data was further filtered using localization probability of 0.7, PEP of 0.05 and having valid values in at least one sample resulted in 2944 class-I glycosylation events derived from 1325 unique proteins in MTBC strains representing lineages 3, 4, 5 and 7. The term “glycosylation event” is used to avoid confusion when a single glycosylation site is glycosylated by more than one type of glycan residues. O-glycosylation constituted 2455 (83%) of the events identified (1311 events at T and 1144 events at S residues) and the remaining 489 sites (17%) were glycosylated at N residues (Fig. 1A,C, Supplementary Table S1). Comparative glycoproteomic analysis among the four MTBC lineages revealed that 945 (32.1%) of the total glycosylation events identified were shared amongst the four lineages (Fig. 1A). Comparison at the level of unique glycoproteins revealed that 44.2% of the glycoproteins were shared among the four lineages, irrespective of the glycosylation site and glycan residues (Fig. 1B).

Among the 57 most common naturally occurring glycan residues, deoxyhexoses (DeoxyHex) was the most frequently identified glycan residue in our search, followed by Hept, pent, Hex, HexN, HexNac/GlcNac, MurNGly and MurNac (Supplementary Table S3). We also identified sugar molecules attached to both ADP and UDP in comparable proportions, which may contribute to an activated nucleotide-sugar substrates for OST (Supplementary Table S3).

The glycosylation events identified per protein ranged from 1 to 33 (Fig. 1D, Supplementary Table S2). The lipoprotein LpqH was found to be the most highly glycosylated protein with no less than 33 events, hosting 24 events on T and 9 events on S residues (Fig. 1D). Notably, all glycan residues found on LpqH were composed of solely hexoses, while a cocktail of glycan residues were observed in other proteins harboring many glycosylation events (Supplementary Table S1). Among the 33 glycosylation events detected on LpqH, 20 events were common across all four lineages. Other highly glycosylated proteins included AcpM, GroeL2, BlaC, DsbF, FadE4 and HupB (Fig. 1D, Supplementary Table S2). Several hypothetical proteins were also glycosylated.

### LpqH and AcpM have glycosylation sites concentrated in the interacting domains

**Figure 2:**
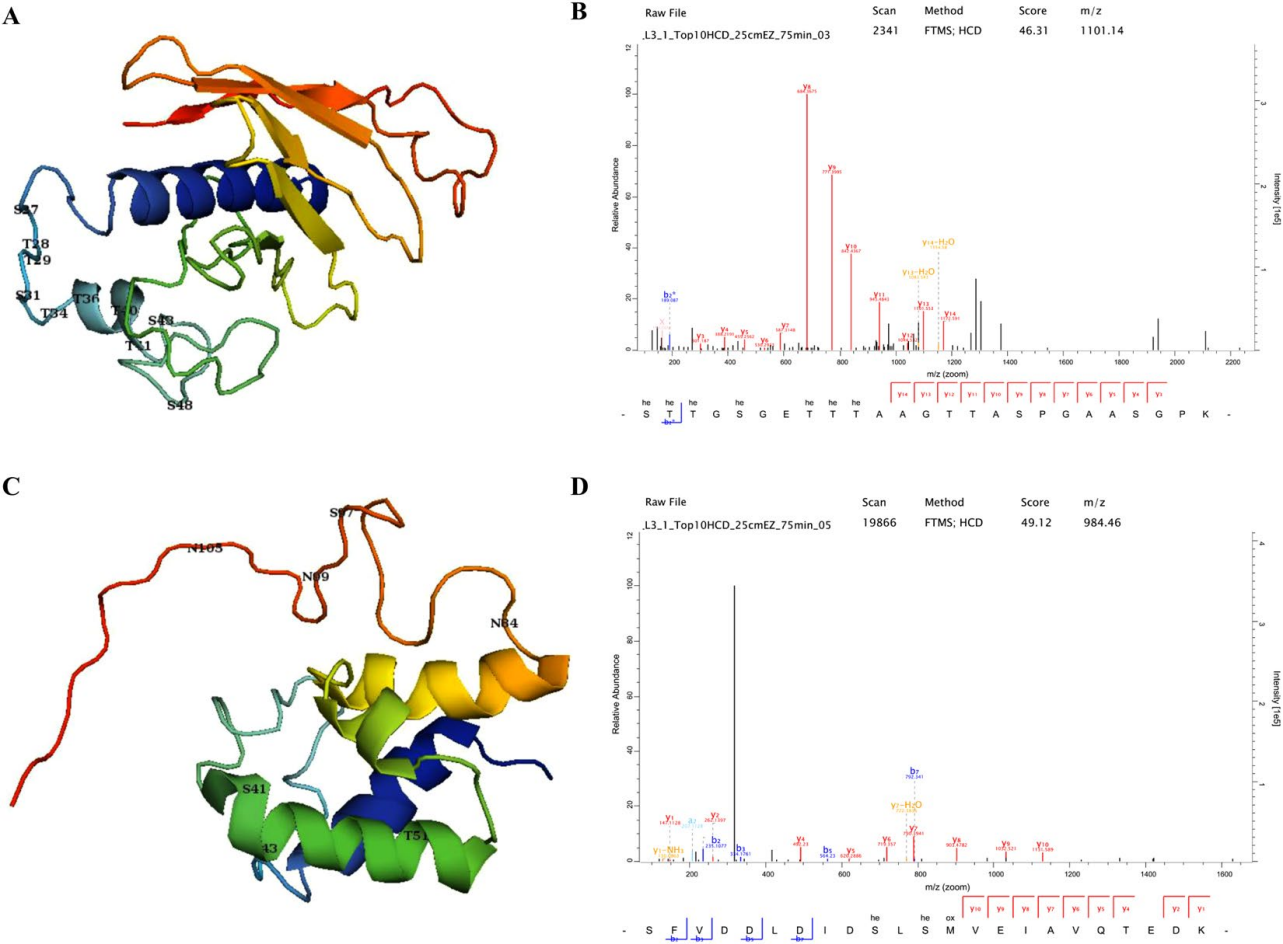
The 3D models, acetylation sites and representative spectra of LpqH (**A**,**B**) and AcpM (**C**,**D**). The glycosylation sites in LpqH are clustered in the N-terminus between residues 27–48 of the 159 amino acid protein (**A**). The four sites, T40, T41, S43 and S48 were located in this binding domain of LpqH. In AcpM, three sites in the helix, S41, S43 and T51, were found in the carrier protein (CP) domain profile (**C**). (he = hexose).

The glycosylation sites in LpqH clustered in the N-terminus, densely located between residues 27–48 of the 159 amino acid protein (Fig. 2A). The amino acid residues from 41–60 are known to be involved in the binding of LpqH with the host MR. The four sites, T40, T41, S43 and Ser48 are located in this binding domain region of LpqH. In AcpM, three glycosylation sites in the helix S41, S43 and T51 are found in the carrier protein (CP) domain profile (Fig. 2C).

### No apparent amino acid sequence specificity for Mtb glycosyltransferases

**Figure 3:**
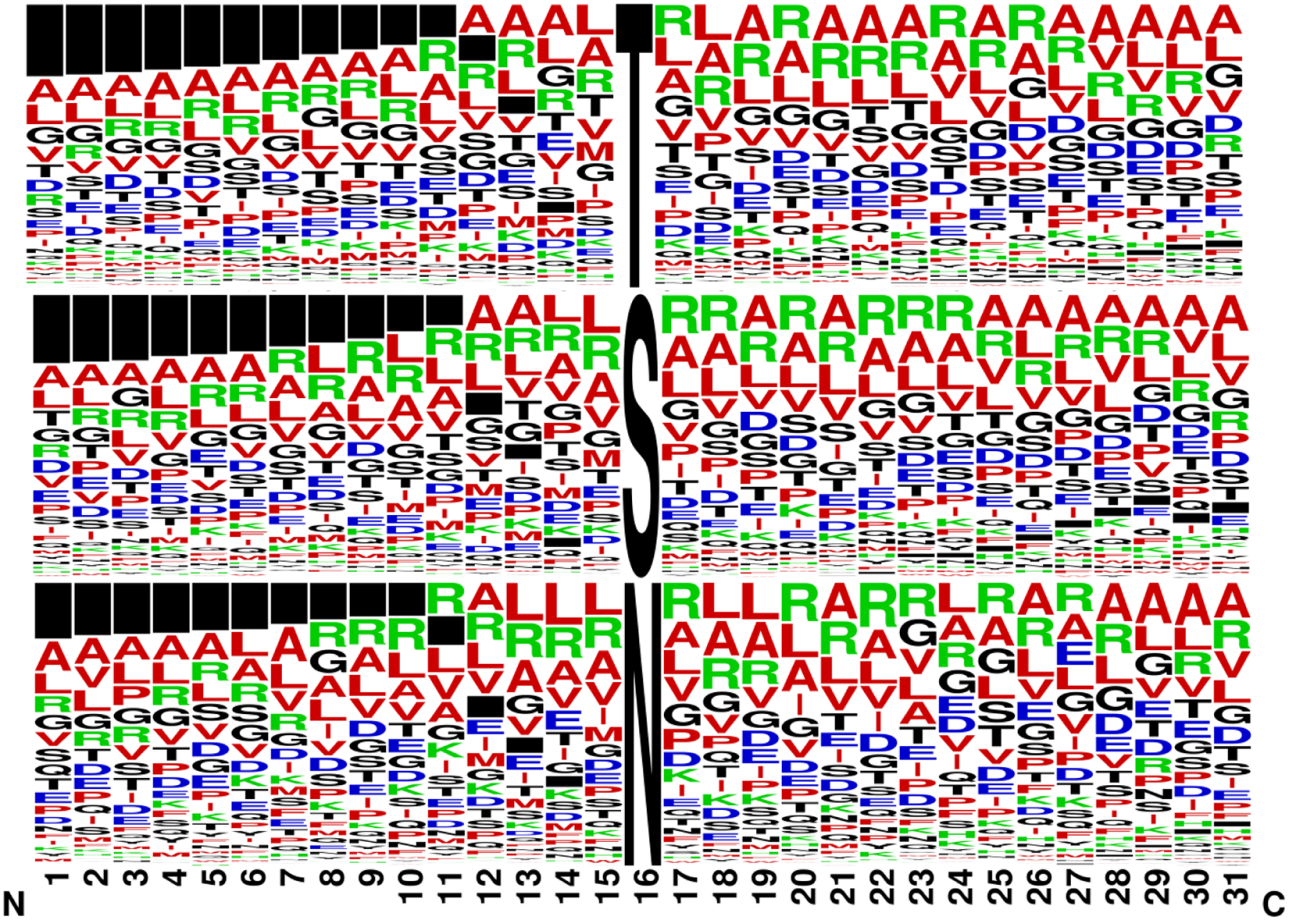
Glycosylation motif analysis. The N- and O-glycosylation motif generated from the high confidence identification indicates a higher likelihood of basic R, hydrophobic P, A, V and L, interspersed with Polar G; and some hydrophilic S and T around the O-glycosylation site. In addition, the glycosylation motifs seem to cluster predominantly at the N-terminus as indicated by the black boxes on the left. The height of each amino acid indicates its relative frequency at that specific position.

Comparing the 31-mer unique sequences of all peptides containing a glycosylation site by WebLogo yielded a “consensus” sequence atlas (Fig. 3). The distribution of the amino acids flanking the modified site showed a relatively high propensity for R, L, A, V, P and G residues (Fig. 3).

### The glycolipoproteins and glycoproteins identified are involved in diverse biological functions

**Figure 4:**
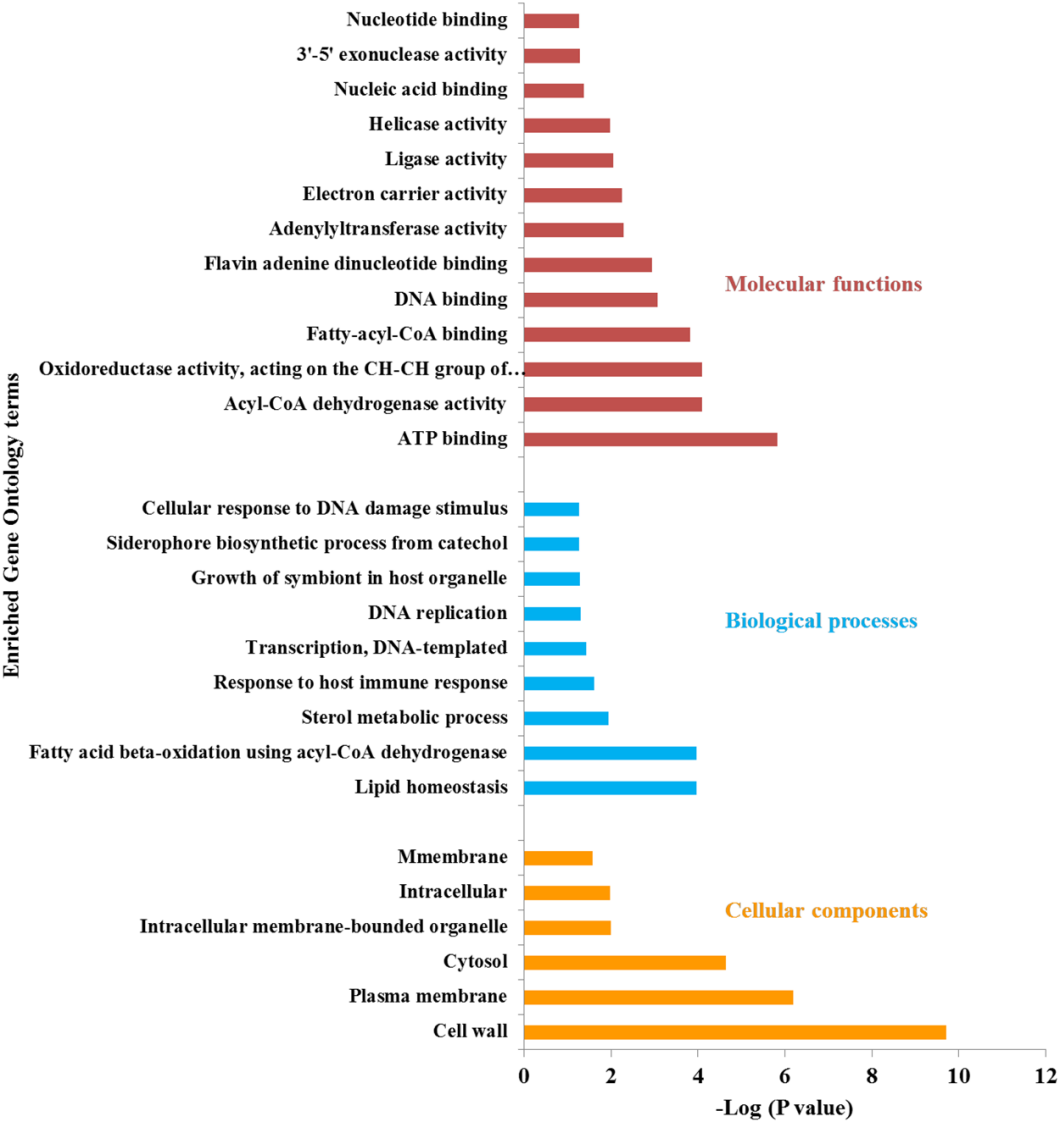
Gene Ontology analysis of Mtb glycoproteins. The gene ontology analysis showed that the majority of the glycoproteins identified were localized in the cell wall and plasma membrane while lipid homeostasis, fatty acid metabolism, and response to the host immune system were among the enriched biological processes.

Based on the Gene Ontology (GO) analysis, fatty acid metabolism and lipid homeostasis, growth of symbiont in the host cell and responses to the host immune system were highly enriched biological processes. The cell wall and the plasma membrane were the two highly enriched cellular components of the glycoproteins identified. The molecular functions include ATP binding, oxidoreductase activity, acyl-CoA dehydrogenase activity, fatty-acyl-CoA binding, helicase activity, DNA binding, electron carrier activity and ligase activity (Fig. 4). The roles of the Mtb glycoproteins identified are summarized in Table 1. The non-glycosylated complement, however, encompassed proteins with functions and localization primarily in the cytoplasm. Furthermore, the GO analysis of uniquely identified glycoproteins provided strain-specific enrichment of biological processes, molecular functions (Supplementary Table S6).

**Table 1:** Virulence-associated membrane-bound glycoproteins, proteins involved in regulation, antimicrobial resistance (AMR) and chaperone proteins identified in MTBC.

Function	Process	Glycoproteins involved
Cell envelope synthesis	Mycolic acid synthesis	AcpM, MmaA1, MmaA2, MmaA3, PcaA, Pks13, FbpB and FbpC
	PDIM synthesis and transport	PpsA, PpsC, PpsD, PpsE, PapA1, FadD26, FadD28, LppX, DrrC and MmpL7
	PG synthesis	PBPs) PbpA (Rv0016c), PbpB (Rv2163c), PonA1 (Rv0050) and LdtA (Rv0116c), MurA, MurE, MurF, LprQ, FtsW, MviN, GlmS, GlmM, DacB1 and Wag31
	Capsule biosynthesis	GlgM (Rv1212c), GlgB (Rv1326c), GlgE (Rv1327c), TreZ (Rv1562c) and MalQ (Rv1781c)
	AG	AftD (Rv0236c), DprE1, EmbC and EmbR
	Lipoglycans (LM, LAM and PI)	PimB (Rv2188c), EmbC, Rv1459c and Rv2181
Membrane transport proteins	Sec	SecA1, SecY, SecD, SecE2, SecF
	Tat	TatB
	MmpL lipid transporters	MmpL1, MmpL3, MmpL4, MmpL5, MmpL6, MmpL8, MmpL9, MmpL10, MmpL11, MmpL12 and MmpL13b
	ATP-binding cassette (ABC)	DrrC, DppA, DppC, DppD, FecB, UgpC, UgpE, ProZ, CydD, MalQ, Rv2326c, Rv2041c, Rv1680, Rv3197, Rv0987, Rv1281c, Rv3092c, Rv1747, Rv1273c, Rv1739c, Rv2564, Rv0073
	Type-VII secretion	EccA1, EccB1, EccCb1, EccA2, EccB2, EccC2, EccD3, EccB3, EccC4, EccB4, EccA5, EccC5,
	Others	CpnT, NanT, IrtA, IrtB, ArsC
MCE family proteins		Mce2A, Mce2D, Mce1E/LprK, Mce3R, Mce1A, Mce4C, Mce1C, Mce3C, Mce2R, Mce1R, Mce1B, Mce2F, Mce2B, Mce3D and Apa*
Regulatory proteins		DevS-DosT/DosR, PhoR, WhiB3, WhiB4, WhiB5, WhiB7, TcrA, PrrA/PrrB, MtrA/MtrB, KdpD, KdpC, MoxR3, NarL, EmbR, PdtaR, GlnB, Mce1R, KstR, BlaR, BlaI and OxyS, Rv1353c, Rv0890c, Rv3095, Rv0494, Rv0043c, RamB, Rv0081, Rv0339c
Chaperones		GroS, DnaK, GroeL1, GroeL2, ClpB, ClpX, Hsp
Role in AMR		BlaC, KatG, RpoC, KasA, AhpD, FadE24, AcpM, IniB, IniC, EthA, OpcA, Wag31, RpoB, EmbR, EmbC, FabG1, RpsL, Mdh, Ndh, Alr, MtrAB, Rv2994, Rv0194, LprG, GyrA and GyrB
Potential drug targets		Mur enzymes, DrrC, PknD, MmpL3, GlgB, GlgE, Hpt, PbpA, PbpB, PonA1 and LdtA

### Glycosylated proteins are involved in 14 specific metabolic pathways

**Figure 5:**
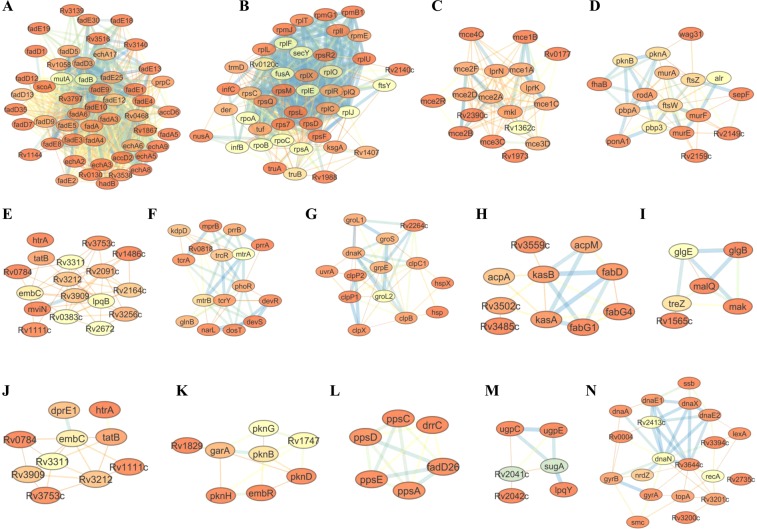
Protein-protein interaction networks of identified glycoproteins generated by Cytoscape. Networks are involved in lipid Metabolism & cell wall synthesis (**A**,**D**,**E**,**H**,**I**,**J**), protein synthesis (**B**), host-pathogen interaction (**C**), chaperone proteins (**G**), regulators (**F**,**K**), phthiocerol dimycocerosate (PDIM) synthesis (**L**), (sugar) transporters (**M**) and DNA replication, repair and recombination (**N**).

Through protein-protein interaction (PPI) network analysis, 14 highly interconnected clusters were identified (Fig. 5). Most of the interacting glycoproteins identified were part of common pathways involved in fatty acid and lipid metabolism, protein synthesis, pathogen-host interaction, PG, AG, mycolic acid and capsule biosynthesis, stress responses, two-component systems (TCS), energy metabolism, and DNA replication repair and recombination (3R) (Fig. 5).

### Glycolipoproteins involved in pathogen-host interaction

Our GO analysis a showed that most of the glycoproteins identified were localized in the cell wall and plasma membrane while lipid homeostasis, fatty acid metabolism, and response to the host immune system were among the biological processes enriched (Fig. 4). Lipoproteins were amongst the highly glycosylated Mtb proteins identified in this study, and are known to be involved in colonization, invasion, evasion of host defence and immunomodulation, cell envelope biogenesis, transport across membrane, nutrient acquisition, adhesion, cell invasion and initiation of inflammatory processes (Table 2)^13,14,36–38^. These glycoproteins include the LpqH, the MCE-family proteins, Apa, Heparin-binding hemagglutinin (HbhA) and LprG.

**Table 2:** Representative biological activities elicited by glycolipoproteins identified from MTBC.

Role or function	Glycolipoprotein(s)
Antigenicity	LpqH, LprG, LppX
Adhesion and cell invasion	LpqH, LprG, LprK, LprN, LppA, LpqG, LppX, MCE
Required for growth	LpqH, LprK, SugA, LpqY, LppY, LpqB
Signal transduction	LprF, LprA, LprG, LppR, LppX, LpqB
Role in AMR	LprG, BlaC
Cell wall metabolism	PbpB, PbpA, PonA1, LprQ, LprK, LppW, LppX, LpqY, LpqB
ACB transport system	UgpE, UgpC, Rv2041c, LpqY, MalQ, DppA, FecB
Degradation	LpqP, LpqI, Rv2672, LpqL
Other enzymes and metabolic activities	GgtB, Rv0526, DsbF, LppZ, LpqD, SodB, Rv0526
Unknown function	Rv3693, Rv0679c, LppG, LpqU, LpqJ, LppO

### Glycosylation of proteins involved in MTBC cell envelope biogenesis

Membrane-associated proteins involved in lipid and fatty acid metabolism, cell envelope biosynthesis, pathogen-host interaction, transport, transcriptional regulation, and chaperone functions were also glycosylated (Fig. 5). After LpqH, the meromycolate extension acyl carrier protein AcpM was the second highly glycosylated protein identified with 21 glycosylation events. AcpM is involved in mycolic acid biosynthesis, a major component of the Mtb cell wall. Other glycoproteins involved in mycolic acid synthesis include methoxy mycolic acid synthases (MmaA1, MmaA2, MmaA3), mycolic acid synthase PcaA, polyketide synthases Pks13, enzymes involved in the synthesis of the Mtb cell wall components (PpsA, PpsC, PpsD, PpsE, PapA1, Rv2951c, FadD26, FadD28, LppX, DrrC and MmpL7), beta-ketoacyl-ACP synthases (KasA, KasB), mycolyltransferases (FbpB and FbpC), mycolic acid biosynthesis a protein FabG1, penicillin-binding glycoproteins (PBPs) (PbpA, PbpB, PonA1 and LdtA), proteins involved in PG biosynthesis (MurA, MurE, MurF, LprQ, FtsW, MviN, GlmS, GlmM, DacB1 and Wag31), AftD, EmbC, enzymes involved in the biosynthesis of alpha-D-glucan (GlgM, GlgB, GlgE, TreZ and MalQ), enzymes involved in biosynthesis of lipoglycans (PimB, Rv2181, MgtA, Ppm1 and Rv1459c).

### Other clinically important glycoproteins identified

Clinically important glycoproteins include BlaC, chaperone proteins, TCS proteins, ESX secretion system proteins and other transporter proteins. Mtb BlaC was glycosylated at 12 sites, while the chaperones GroEL2 and GroS were found to have 18 and 10 glycosylation sites, respectively.

### Glycosylation of cytoplasmic proteins in MTBC

Cytoplasmic proteins involved in translation and DNA metabolism were glycosylated (Fig. 5A,B). The cytochrome P450 proteins were also glycosylated.

### MTBC strains exhibit lineage-specific glycoproteomic profiles

**Figure 6:**
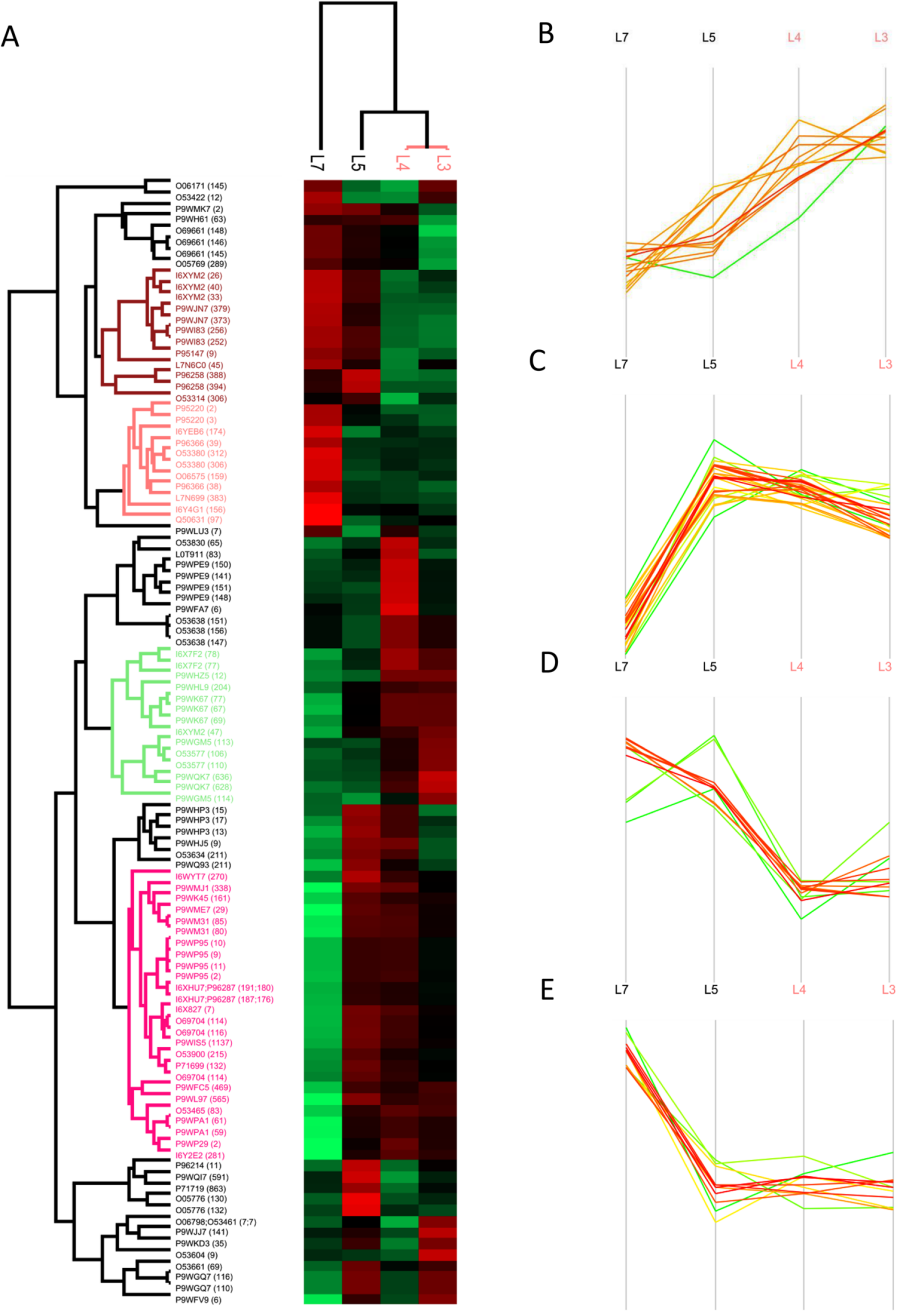
Profile of the 101 differentially glycosylated proteins in MTBC. Hierarchical clustering of differentially glycosylated proteins (**A**), proteins hyper-glycosylated in lineage 3 and lineage 4 strains (**B**), proteins hyper-glycosylated in lineage 3, lineage 4 and lineage 5 strains (**C**), proteins hyper-glycosylated in lineage 5 and lineage 7 strains (**D**), proteins hyper-glycosylated in lineage 7 strain (**E**).

The GO analyses of exclusively identified glycoproteins provided strain-specific enrichment of biological processes and molecular functions (Supplementary Table S6). Among the 2944 glycosylation events detected, 1010 had valid values in at least six valid LFQ intensity values from the total of 12 biological replicates (50%) and were thus subjected to further quantitative analysis. The missing values were imputed from the normal distribution and the log2-transformed data was normalized to Z-scores for further statistical testing. Multiple sample test analysis at a P < 0.05 level of significance revealed that 101 sites on 67 proteins were differentially glycosylated (differential abundance of a peptide/protein glycosylated at a specific site) between the four MTBC lineages studied (Fig. 6A, Supplementary Tables S4 and S5). Notably, most of the differentially glycosylated proteins (43/67: 64%) were located in the cell wall and cell membrane or possess a membrane component. These proteins belonged to different functional categories including lipid metabolism, cell wall and cell processes, virulence, detoxification and adaptation, and hypothetical proteins (Supplementary Table S5). Clinically important differentially glycosylated proteins include the lipoarabinomannan carrier protein LprG, chaperone proteins GroEL1, class A β-lactamases BlaC, mammalian cell entry (Mce)-family protein Mce2D, peroxidase BpoB, penicillin-binding glycoprotein PbpB, and a number of proteins involved in fatty acid and lipid metabolism (Supplementary Table S5).

In the hierarchical clustering of the differentially glycosylated proteins, the modern lineages (lineage 3 and lineage 4 strains) clustered together, separated from the ancient lineages (lineage 5 and lineage 7 strains) (Fig. 6A). Four separate clusters of proteins with a particular glycosylation profile were generated. The first cluster included 14 hyper-glycosylated proteins (having a significantly higher number of glycosylation events at a specific position in a protein than the average) in lineage 3 and lineage 4 strains, encompassing LppW (S67, N69, T77), UvrA (T628, N636), PurK (S204), PPE42 (S12), FadE35 (S106, S110), LdtA (T147, S151, S156), DsbF (T47), NarL (S113, T114) and Rv3483c (T77, S78) (Fig. 6B). Cluster two included 26 glycoproteins such as Mce2D (S270), DagK (S2), LprG (N161), UvrC (T469) and VapC45 (T83) that were hyper-glycosylated in lineage 3, lineage 4 and lineage 5 strains (Fig. 6C). Membrane proteins DsbF (S26, T33, T40) and Rv0412c (388, 394), Mrp (S373, T379), ATPase MoxR3 (S306) and PknA (T252, N256) were hyper-glycosylated in ancient lineages (lineage 5 and lineage 7 strains) (Fig. 6D). Cluster four included 11 glycoproteins, such as FadD9 (T97), DacB1 (T306, T312), FadD34 (T383), short chain type dehydrogenase/reductase Rv2766c (174) and peroxidase BpoB (N159), that were hyper-glycosylated in lineage 7 strains (Fig. 6E). Virulence factors such as PbpB (S83), GroeL1 (T141, T148, S150, S151), VapC10 (S6), VapB11 (S7) and the transcriptional regulatory protein Rv0818 (S65) were hyper-glycosylated in lineage 4 strains. The alanine rich protein Rv3863 (S11), the transmembrane ATP-binding protein ABC transporter protein Rv2326c and a polyketide synthase involved in sidrophore biosynthesis MbtD (T591) were hyper-glycosylated in lineage 5 strains (Supplementary Table S4).

## Discussion

Our analysis identified with high confidence a total of 2944 glycosylation events on 1325 Mtb unique proteins. To our knowledge, the discovery of such a large number of glycosylation sites in these four clinical strains from different MTBC lineages is unprecedented. About 83.4% of the glycosylation events were localized on S and T residues, indicating a possible interplay with phosphorylation. It has been reported that different glycosylation events may occur on the same S and T residues of the protein or competitively at adjacent or residues in close proximity, and hence potentially allow control of cellular signaling^27^. The study provides the first evidence on N-linked protein glycosylation in Mtb and Gram-positive bacteria. Protein glycosylation occurred at numerous sites on surface-exposed proteins with no apparent amino acid sequence specificity (Fig. 3)^7^. As previously reported, there is a relatively high propensity for R, A, P, L, G, V, S and T flanking the modified sites in a significant portion of the glycosylation sites mapped^11,27,30^ (Fig. 3). However, a number of suggested signature motifs were identified in nearly 17% of the events and R was enriched between the −8 and +8 positions in contrast to the D/E-Y-N-X-S/T (Y, X #P) motif proposed for N-glycosylation^24^. This difference might partly be due to the diversity of the glycan residues analyzed and the high degree of specificity for both their donor and acceptor substrates in the glycosyltransferases^39^. Comparative analysis revealed that only 32.1% of the glycosylation events and 44.2% of the glycoproteins were shared among the four lineages. The higher versatility at the level of PTMs may indicate the power of PTMs in explaining the phenotypic variability among MTBC than the proteomic studies.

DeoxyHex was the most frequently identified glycan residue in our search, followed by Hept, pent, Hex, HexN, HexNac/GlcNac, MurNGly and MurNac (Supplementary Table S3). In bacteria, 6-deoxy-hexoses, like fucose and rhamnose, are important components of cell surface glycans^40^. The pentose sugars arabinose and galactose are components of the heteropolysaccharide, AG, which serves to connect PG with the outer mycolic acid layer^19^. Bacterial heptosyltransferases are reported to be involved in O-glycosylation of autotransporters using ADP-heptose^41^. The presence of frequently occurring glycan residues attached to lipoproteins, extracellular polysaccharides (EPSs) and glycoproteins might alter the structure and function of these biomolecules in particular and bacterial physiology in general^40^. We identified both ADP- and UDP linked to different sugar molecules to form an activated nucleotide-sugar substrates for OST (Supplementary Tables S1 and S3). Most publications reported that only UDP-linked sugars were the substrates for OST^8,22^, while other reports showed that a particular OST, heptosyltransferase, used ADP-heptose as an activated nucleotide-sugar substrate^41^.

The outermost layer of the Mtb cell envelope is a major determinant of virulence and pathogenicity, and is mainly composed of proteins, polysaccharides and small amount of lipids^12,42^. It acts as a permeability barrier of the cell envelope, promoting the phagocytosis of Mtb^43^, maintaining cell integrity, regulating phagosome maturation^44^ and playing diverse roles in the pathogen-host interactions^42,43^. The gene ontology analysis showed that the majority of the glycoproteins identified were localized in the cell wall and plasma membrane while lipid homeostasis, fatty acid metabolism, and response to the host immune response were among the biological processes enriched. Besides, the PPI network analysis showed that most of these cell-envelope associated glycoproteins are involved in pathogen-host interaction and fatty acid/lipid metabolism. These cell envelope-associated glycoproteins have been shown to have a vital role in Mtb virulence and pathogenesis (reviewed in^12^).

Lipoproteins were amongst the highly glycosylated Mtb proteins identified in this study. Lipoproteins are a functionally diverse class of membrane-bound proteins involved in colonization, invasion, evasion of host defence and immunomodulation, cell envelope biogenesis, transport across the membrane, nutrient acquisition, adhesion, cell invasion and initiation of inflammatory processes (Table 2)^13,14,36^. The lipoprotein LpqH was the most densely glycosylated lipoprotein detected, with 33 N-terminally clustered O-glycosylation events, where all glycan residues were hexoses (Supplementary Table S1). Notably, these sites were densely located between residues 27–48 within the 159 amino acid protein. Some of the glycosylation sites have previously been reported as part of the MR binding domain of LpqH, as shown in the 3D model (Fig. 2)^30,45^. Three of the sites, T41, S43 and S48, were part of a mature protein fragment (residues 41–60) that was reported to prevent uptake of Mtb by macrophage-like U937 cells^46^. Altering the glycosylated Ser residues in LpqH have been shown to affect binding affinity and exposure to proteolytic cleavage^10^. LpqH, an immunodominant TLR2 agonist, is crucial for Mtb growth and multiplication in IFN-γ-activated macrophages as well as in IFN-γ-deficient mice^47^. Mannosylated LpqH is the major adhesin for the macrophage MR and DC-SIGN, and the mannose residue serves as an adhesin for binding to the host MR^29^.

Other groups of identified glycolipoproteins involved in pathogen-host interaction are the MCE-family of proteins^36^. These glycoproteins have an active role in disease development and in-host virulence^12^. A total of 14 glycosylation events were identified on proteins expressed from the four Mtb *mce* operons (*mce1*, *mce2*, *mce3* and *mce4*). The invasion-/adhesin-like MCE family glycolipoproteins encoded by *mce*s are located at the cell surface of Mtb and possibly involved in entry and survival inside macrophages^48^.

A number of other clinically important glycoproteins were identified. The cell surface glycoprotein Apa binds to DC-SIGN and surfactant protein, facilitates colonization and invasion of host cells^49^. Changes in the glycosylation pattern of Apa lead to a reduced stimulatory T-lymphocyte response, exhibiting the biological role of the glycan moiety^50^. Glycosylation is also required for proper localization of superoxide dismutases (SodB)^51^. The immunogenic glycoproteins MPT64 and Apa are virulence factors involved in Mtb infection of human cells and is a promising candidate for a subunit-based anti-TB vaccine^12,52^. Heparin-binding hemagglutinin (HbhA) glycoprotein mediates adherence to epithelial cells and is required for extrapulmonary dissemination of Mtb^53^. The lipoprotein LprG is another glycolipoprotein that blocks host cell phagosome-lysosome fusion, and is required for full Mtb virulence^54^.

Glycoproteins associated with drug efflux pumps, drug-hydrolyzing enzymes, or capable of altering Mtb cell wall permeability mediates the development of AMR (reviewed in^12^). These include proteins like the mycobacterial membrane protein large (MmpL) proteins, daunorubicin-dim-transport integral membrane protein ABC transporter (DrrC), class a beta-lactamase (BlaC) and LprG (Table 1). DrrC, Rv0194, Rv2994, Rv1273c and a number of MmpL glycoproteins are efflux pumps for anti-TB drugs, contributing to AMR^55,56^. In addition to a role in drug resistance, MmpLs are involved in the export of cell wall associated lipids and siderophores, and are attractive pharmacological targets^57,58^. BlaC hydrolyzes nitrocefin and other β-lactams, thereby increasing Mtb resistance towards different classes of β-lactam antibiotics^4^. LprG controls cell wall permeability and efflux of drugs, and therefore plays a role in Mtb susceptibility to first-line anti-TB drugs^5^.

The study identified a number of membrane-associated glycoproteins involved in cell envelope biosynthesis and drug efflux pumps, which are potential Mtb drug targets (Table 1, Fig. 5). AcpM was second most densely glycosylated protein involved in mycolic acid biosynthesis, one of the major components of the Mtb cell wall. Glycosylation sites Ser41, Ser43 and Thr51 were detected within the AcpM CP domain profile. Importantly, one of the glycosylation sites identified (Ser41) is the binding site for 4′-phosphopantetheine, an activator of AcpM^59^. Other glycoproteins involved in mycolic acid synthesis include MmaA1, MmaA2, MmaA3 and PcaA, Pks13, KasA, KasB and FabG1. Bacilli lacking all mycolic acid methyltransferases are viable but highly attenuated and hyperinflammatory in mice^60^. Pks13 catalyzes the last condensation step of mycolic acid biosynthesis and is essential for the mycobacterial survival^61^. Glycoproteins FbpB (Ag85B) and FbpC (Ag85c) also possess a mycolyltransferase activity^62^. These glycoproteins help to maintain the Mtb cell wall integrity by catalyzing the transfer of mycolic acids to cell wall AG, and through the synthesis of the virulence factor cord factor (trehalose 6,6′-dimycolate, TDM)^62^. Furthermore, FbpB and FbpC are T- and B-cell antigens and may have an application in sero-diagnostics^63^.

Penicillin-binding glycoproteins (PBPs) PbpA, PbpB, PonA1 and LdtA are transpeptidases involved in the synthesis of cross-linked PG that is part of the cell wall biogenesis^64^. Other essential glycoproteins involved in PG biosynthesis include MurA, MurE, MurF, LprQ, FtsW, MviN, GlmS, GlmM, DacB1 and Wag31^65^. Glycoproteins involved in PG biosynthesis, such as Mur enzymes and PBPs, are potential antibiotic targets^65^. Alpha-(1 → 3)-arabinofuranosyltransferase (AftD) is involved in the biosynthesis of the AG region of the mAGP complex, an essential component of the mycobacterial cell wall^19^. EmbC is involved in the polymerization of arabinose into the arabinan of the mycobacterial cell wall AG and is linked to resistance to EMB^66^.

Polyketide synthases (PpsA, PpsC, PpsD, PpsE), PapA1, Rv2951c, FadD26 and FadD28 are multifunctional enzymes involved in the synthesis of the Mtb cell wall component, PDIM and other lipids^67^, while the glycolipoproteins LppX, DrrC and MmpL7 are required for the translocation and localization of PDIM in the cell wall^68^. PDIM comprise of a number of virulence-enhancing lipids that act as defensive, offensive, or adaptive effectors of virulence^69^. Inactivation of mycobacterial *pps* and *drr* operons has been linked to defects in PDIM synthesis and secretion, respectively^70^. PknD, a regulator of MmpL7, has been proposed to be a potential anti-TB drug target^71^.

Glycosyltransferases such as GlgM, GlgB, GlgE, TreZ and MalQ are enzymes involved in the biosynthesis of alpha-D-glucan, a constituent of Mtb capsular polysaccharides with D-arabino-D-mannan (AM) and D-mannan^1,42^. These enzymes are required for Mtb virulence^72^. GlgE-mediated 1,4 α-glucan synthesis has been implicated in *in vitro* lysosomal stress and can potentially be exploited for killing intracellular Mtb^73^. The Thr10 glycosylation site in GlgE has been shown to be a regulatory kinase substrate and a validated anti-TB drug target^74^. GlgB is a potential target for inhibitors^75^. Glycosylated mannosyltransferases PimB and Rv2181 are involved in the biosynthesis of lipoglycans LM, LAM and phosphatidylinositol (PI)^76^. Mannosyltransferases MgtA, Ppm1 and Rv1459c are involved in the synthesis of immunomodulatory LM and LAM via alpha-(1 → 6)-mannopyranosyltransferase activity^77^. A number of glycosylated fatty acyl-AMP ligases that have been shown to play a role in cell wall biosynthesis, production of complex lipids and growth^78^ were identified. As discussed above, glycosylation is involved in regulating the activity of different enzymes. In this study, identification of glycosylated glycosyltransferases (with rare abundance) may play a role in regulating its function as an enzyme^79^. There are reports on auto-glycosylation mediated activation of glycosyltransferases in eukaryotes^80–82^.

Other clinically important glycoproteins identified include chaperone and TCS proteins. The differential expression of chaperone glycoproteins, such as GroeL2 and GroS, in response to heat shock have previously been reported^12^. TCS regulate various aspects of mycobacterial physiology, including virulence, dormancy, persistence, and drug resistance^83^. The glycoprotein PhoPR regulates multiple virulence-associated processes in Mtb, including the biosynthesis of polyketide-derived lipids and acyltrehaloses. The inactivation of acyltrehaloses attenuates Mtb sufficiently to make it a possible live vaccine candidate^12,16^. The DosR/WhiB3 regulon is associated with hypoxia and redox adaptation, while WhiB3/PhoP is involved in cell wall lipid biosynthesis^84^. The DevS/DosR regulon is required for full Mtb virulence and is involved in regulating stress, dormancy and hypoxia^85^.

Twenty glycosylation events on proteins belonging to the specialized ESX secretion system components, including the crucial T-cell antigen ESAT-6, were detected. The ESX secretion system is essential for full Mtb virulence (ESX-1) and physiological processes (ESX-3)^86^. Five proteins involved in the general secretion (Sec) pathway and a twin-arginine translocation pathways (TatB) were also found to be glycosylated. These specific proteins are essential for bulk export of proteins in Mtb^86^. CpnT, the first autotransporter-like protein to be identified in Mtb, was glycosylated at a domain that is required for the membrane localization of this protein^87^. Our former study showed that glycoproteins including LpqH, AcpM, GroEL1, GroEL2, DnaK, Pks13, KatG, LprK, SecA1 and a number of proteins involved in lipid metabolism and protein synthesis were highly acetylated in Mtb^88^, which might indicate the interaction among different PTMs in fine-tuning specific cellular processes.

A recent report demonstrated a mechanism for co-regulation of Mtb cell wall synthesis and ribosome maturation (protein synthesis), and hence glycosylation of proteins involved in these two processes (Fig. 5A,B) may have a regulatory role^89^. Evidence for glycosylation of DNA-binding proteins (Dps) has been observed in *Salmonella enterica* in response to starvation and/or oxidative stress^90^. This is the first report on glycosylation of those cytosolic proteins. Glycosylation of cytochrome P450 has been demonstrated in eukaryotes (CYP2W1)^91^ and in viral cytochrome P450 (YP_143162)^92^. Glycosylation in this regard may enable the proper localization of cytochrome P450^14,93^. Cytochrome P450 plays a role in steroid metabolism, drug deactivation, fatty acid metabolism, xenobiotic detoxification and catabolism of exogenous compounds as a source of energy^94^. Fatty acid metabolism is a major source of carbon and energy in Mtb^95^.

The PTMs identified by *in vitro* culture may only reflect the mycobacterial phenotype in the absence of stress, which may not completely if at all overlap patterns during infection. So, further mapping the exclusive presence and/or differential abundance of Mtb glycoproteins naturally or during exposure to environmental stress or infection may contribute to elucidate the selective advantages and survival strategies adopted by a specific pathogen. This information is fundamental for any drug or vaccine discovery process^12^. More than 64% of the differentially glycosylated proteins were found to be cell envelope-associated proteins. These glycoproteins are reported to be involved in Mtb virulence and pathogenesis (reviewed in^12^). The hierarchical clustering of the differentially glycosylated proteins coincided with the phylogeny among the MTBC, where the modern lineages (lineage 3 and lineage 4 strains) clustered together, separated from the ancient lineages (lineage 5 and lineage 7 strains) (Fig. 6A)^96^.

Clinically important proteins such as FadE35, LppW, LdtA, PurK, PPE42 and UvrA were hyper-glycosylated in lineage 3 and lineage 4 strains compared to the ancient lineages^64^. PurK has been identified to be a high-confidence drug target^97^. The antigen PPE42 is known to elicit humoral immune response against Mtb^98^. Glycoproteins including LprG, Mce2D, DagK, UvrC and VapC45 were hyper-glycosylated in all lineages except lineage 7 strains. LprG plays a role in transport and localization of the TLR2 agonists, LAM, PIM, LM and triacylglycerides to the cell surface, maintaining cell envelope integrity, and inhibition of phagosome-lysosome fusion, thereby enhancing Mtb survival inside macrophages^5,54^. The DagK is involved in the biosynthesis of Mtb virulence factors PI and PIMs^99^. The membrane proteins DsbF and Rv0412c, iron-sulfur cluster carrier protein Mrp, ATPase MoxR3 and PknA were hyper-glycosylated in lineage 5 and lineage 7 strains. A number of proteins involved in lipid metabolism such as FadD9, FadD34, and PG synthesis like DacB1, and oxidoreductases BpoB and Rv2766c, were hyper-glycosylated in lineage 7 strains. Four glycosylation sites on GroeL1, a chaperone involved in mycolic acid biosynthesis during biofilm formation^100^, were uniformly hyper-glycosylated in lineage 4 strains. Penicillin-binding membrane protein PbpB, another hyper-glycosylated lineage 4 strains, is an essential enzyme involved in peptidoglycan biosynthesis and has been predicted to be an important drug target^101^. These proteins are essential virulence factors used by Mtb for cell wall biosynthesis, stress response, immunomodulation, efficient host cell invasion, survival, growth and other physiological processes^102^. The relative abundance of these essential glycoproteins across the different lineages of Mtb might lead to a specific phenotype with better adaptability to the host.

Identification of glycoproteins and their function contributes to a better understanding of the pathogenesis and survival strategies adopted by Mtb. This knowledge is fundamental for diagnostic, drug or vaccine discovery process. Many anti-TB drugs target the biosynthesis of PG, MA and AG, drug efflux pumps and other virulence factors used by Mtb to efficiently invade and multiply inside the host^12^. Our study has identified a large number of membrane-associated glycolipoproteins involved in Mtb pathogenesis. We present a comprehensive glycoproteome map of Mtb and show that there are significant quantitative differences across the various Mtb lineages that may directly influence phenotype. Further purification and detailed functional studies addressing selected uncharacterized glycoproteins may reveal the physiological role of protein glycosylation in defining the phenotype of a bacillus. These findings expand the current understanding of the nature and diversity of Mtb glycoproteins, open a new avenue of research for identification of potential drug targets, and create opportunities to engineer glycoproteins for their clinical applications^103^.

## Methods

### Mtb strains and growth conditions

Four clinical strains representing four different Mtb lineages, lineage 3 (CAS-DELHI), lineage 4 (FSP471.1), lineage 5 (*M*. *africanum*) and lineage 7 (*Aethiops vetus*^104^) strains, were cultured on Middlebrook 7H10 agar plates for 32 days. The details of culturing, sample handling and inactivation were performed as previously described in Yimer *et al*.^105^.

#### Proteomic analyses

(i) **Preparation of cell lysates**. The Mtb cell pellets were mechanically disrupted by bead beating with a MagNa Lyser (Roche, US) as described by Yimer *et al*.^105^.
(ii) **In-gel trypsin digestion**. Gel-fractionated protein samples (100 µg) from Mtb cells grown to late exponential phase were stained using a Colloidal Blue Staining kit (Invitrogen, CA) and each gel-lane was divided into six fractions. Each fraction was subjected to in-gel reduction, alkylation, and tryptic digestion as previously described^106^. Proteins were reduced using 10 mM DTT, alkylated with 55 mM iodoacetamide and digested with sequence grade trypsin (Promega, 1:100; w/w) overnight at 37 °C in 50 mM NH_4_HCO_3_. The in-gel digested protein samples were extracted using 50% and 100% acetonitrile (ACN), dried by SpeedVac concentrator (Eppendorf, concentrator 5301) and re-suspended using 0.05% trifluoroacetic acid (TFA). The extracted protein samples were purified using C_18_ stage tips by stacking three discs from Empore and transferred to auto-sampler nano LC vials for LC-MS/MS analysis as.
(iii) **Nano-LC-MS/MS analysis**. Peptide characterization and quantitation were performed by nano LC*-*MS*/*MS using a Q Exactive™ Hybrid Quadrupole-Orbitrap™ Mass Spectrometer interfaced with an EASY1000-nano-electrospray ion source (Thermo*-*Fisher Scientific, Biberach, Germany). The LC gradient was from 2% to 90% solvent B (0.1% FA in 97% CAN) in 50 µm × 15 cm analytical columns (PepMap RSLC, C18, 2 µm, 100 Å, Thermo Scientific) for 75 min analysis at a flow rate of 0.3 μl/min. The mass spectrometer was operated in data-dependent acquisition mode with automatic switching between MS and MS/MS scans.
  The full MS scans were acquired at 70K resolution with automatic gain control (AGC) target of 1 × 10^6^ ions between m/z = 300 to 1800 and were surveyed for a maximum injection time of 200 milliseconds (ms). Higher energy collision dissociation (HCD) was used for peptide fragmentation at normalized collision energy set to 28. The MS/MS scans were performed using a data-dependent top10 method at a resolution of 17.5K with an AGC of 5 × 10^4^ ions at maximum injection time of 100 ms and isolation window of 2.0 m/z units. An underfill ratio of 10% and dynamic exclusion duration of 30 s was applied. For each Mtb lineage, three biological replicates were analyzed with each biological replicates fractionated into six gel bands, resulting in a total of 72 analytical runs (four lineages * three biological replicates * six fractions).
(iv) **Protein and PTM identification**. The Maxquant software (version 1.5.7.4) was employed for protein and glycosylation site identification from the raw MS data^107^. The raw mass spectral data were searched against the Uniprot Mtb protein database containing 3993 protein sequences concatenated to reverse decoy database and protein sequences for common contaminants. Trypsin [KR].[^P] was specified as a cleavage enzyme with up to two missed cleavages. The “re-quantify” and “match between runs” options were utilized with a retention time alignment window of three min. Carbamidomethylation of cysteine residues was specified as a fixed modification and acetylation on protein N-terminal, conversion of N-terminal glutamine and glutamic acid to pyroglutamic acid, and oxidation of methionine were set as the variable modifications.
  For the PTM analysis, a number of glycan residues were configured in the MaxQuant search at three different amino acid residues, N, S and T (Supplementary Table S3) and were set to variable modification. Both unique and razor peptides were used for the quantification of PTM abundance. Peptides with a minimum length of seven amino acids and detected in at least one or more of the replicates were considered for identification. For protein identification, a minimum of two peptides, of which at least one was unique, was required per protein group. All other parameters in MaxQuant were set to default values.
(v) **Bioinformatics analysis.**

### Statistical analysis

Statistical significance was determined with multiple-sample test at a *P* < *0*.*05* level of significance using Perseus software (version 1.6.0.7). All modified peptide spectra were validated by applying stringent site localization probability of >0.70 and PEP of <0.05 prior to further analysis. PTM sites with a minimum of one valid value from the total samples were considered for PTM site identification. Modified peptides with valid values in at least 50% of the samples were considered for label-free relative quantification analysis.

### Analysis of N- and O-glycosylation motifs

A sequence logo generators WebLogo (http://weblogo.berkeley.edu/logo.cgi) was used to identify the enriched amino acid motifs flanking the glycosylated sites. The sequence windows from the identification table were used to generate the sequence motifs for the three modified amino acids S, T and N.

### Gene Ontology analysis of glycosylated proteins

The biological processes, cellular component and molecular function for the identified glycoproteins were analyzed using DAVID Bioinformatics Resources 6.7. The proteins were classified by GO annotation based on three terms; molecular function (MF), biological process (BP) and cellular component (CC).

### Protein-protein interaction network analysis

Protein-protein interaction (PPI) networks were generated via STRING database version 10 with a high confidence threshold of 0.7 and imported into Cytoscape software (version 3.5.0) to produce the final interaction networks. Highly interconnected clusters were identified using MCODE and ClusterOne plug-in toolkits.

## Supplementary information

Supplementary Dataset S1-S6

## Data Availability

The mass spectrometry proteomics data have been deposited to the ProteomeXchange Consortium via the PRIDE partner repository with the dataset identifier PXD009676.
